# Untreated Primary Hypothyroidism Manifesting as Cardiac Tamponade

**DOI:** 10.7759/cureus.61169

**Published:** 2024-05-27

**Authors:** Narain Badhey, Tarini Salvaji, Hemanth Badhey

**Affiliations:** 1 Cardiology, Touro College of Osteopathic Medicine, New York, USA; 2 Surgery, Bhaskar Medical College, Hyderabad, IND; 3 Cardiology, St. Francis Hospital, Roslyn, USA

**Keywords:** noninvasive cardiovascular imaging using cardiovascular ultrasonography, echocardiogram, diastolic collapse, cardiovascular collapse, hypothyroid myxedema coma, hypothyroidism, pericardial window, hypothyroid pericardial effusion, cardiac tamponade, hypothyroidism cardiovascular manifestations

## Abstract

Hypothyroidism is a condition characterized by low thyroid hormone levels that can affect multiple organ systems with varying symptomatology. Common cardiac manifestations of hypothyroidism include bradycardia and decreased cardiac output. Pericardial effusion can also occur as a result of the condition and rarely can progress to cardiac tamponade. Patients with cardiac tamponade occurring as a result of underlying hypothyroidism can present atypically compared to those experiencing cardiac tamponade due to other causes. Patients with cardiac tamponade as a result of underlying hypothyroidism may present as minimally symptomatic with stable vital signs. Close monitoring of patients with pericardial effusions with underlying hypothyroidism is essential to permit early diagnosis and treatment of the condition. We outline the case of a 73-year-old male presenting with cardiac tamponade due to underlying hypothyroidism necessitating an urgent pericardial window.

## Introduction

Hypothyroidism is a common endocrine disorder that can affect multiple organ systems with diverse clinical manifestations and wide ranges of severity [1]. The total prevalence of the condition and the prevalence of untreated hypothyroidism are increasing [2]. Hypothyroidism disproportionately affects women and most commonly presents as fatigue, lethargy, cold intolerance, weight gain, or constipation [1,3]. Cardiac manifestations include decreased cardiac output, bradycardia, and impaired cardiac muscle relaxation [4]. While pericardial effusion is another known consequence of hypothyroidism with incidence estimated between 3% and 37%, progression to cardiac tamponade is rare [5].

We report a case of a 73-year-old male with primary hypothyroidism presenting with circumferential pericardial effusion and cardiac tamponade necessitating a pericardial window.

## Case presentation

**Figure 1:**
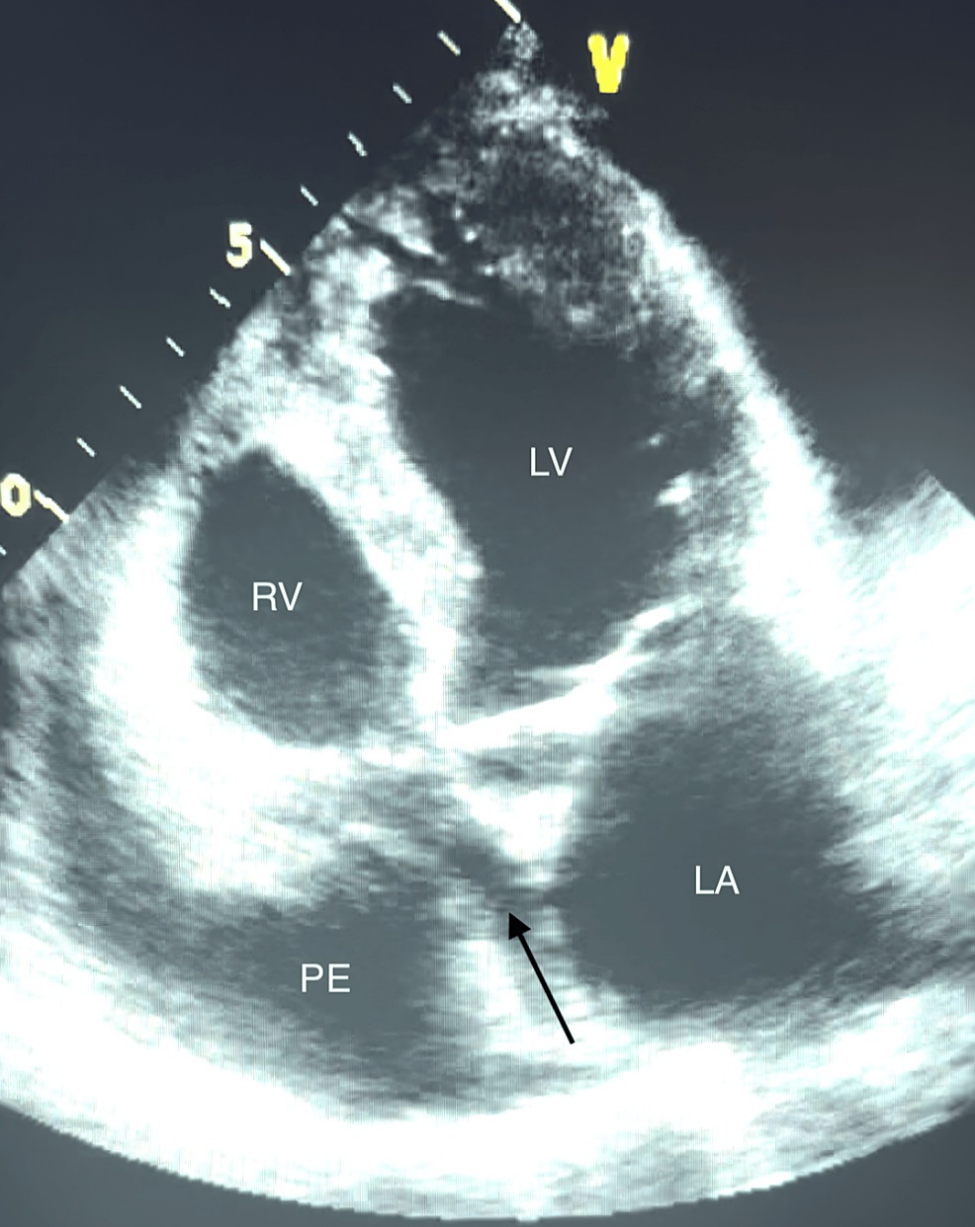
Apical view on TTE revealing pericardial effusion causing right atrial collapse The arrow denotes the collapsed right atrium as a result of circumferential pericardial effusion. LA: left atrium; LV: left ventricle; PE: pericardial effusion; RV: right ventricle; TTE: transthoracic echocardiogram

**Figure 2:**
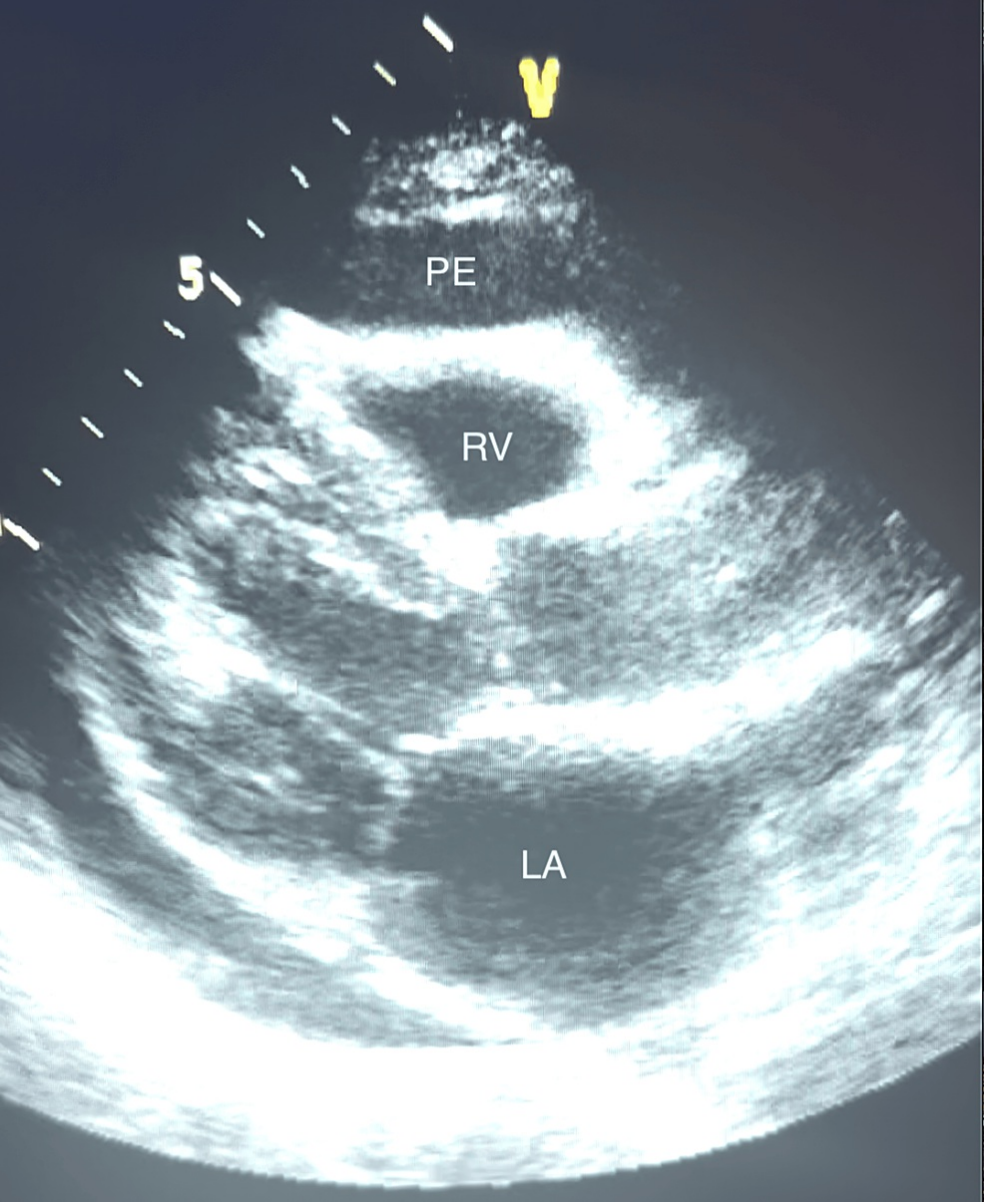
Parasternal long-axis view on TTE revealing right ventricular collapse as a result of pericardial effusion LA: Left atrium; PE: pericardial effusion; RV: right ventricle; TTE: transthoracic echocardiogram

**Figure 3:**
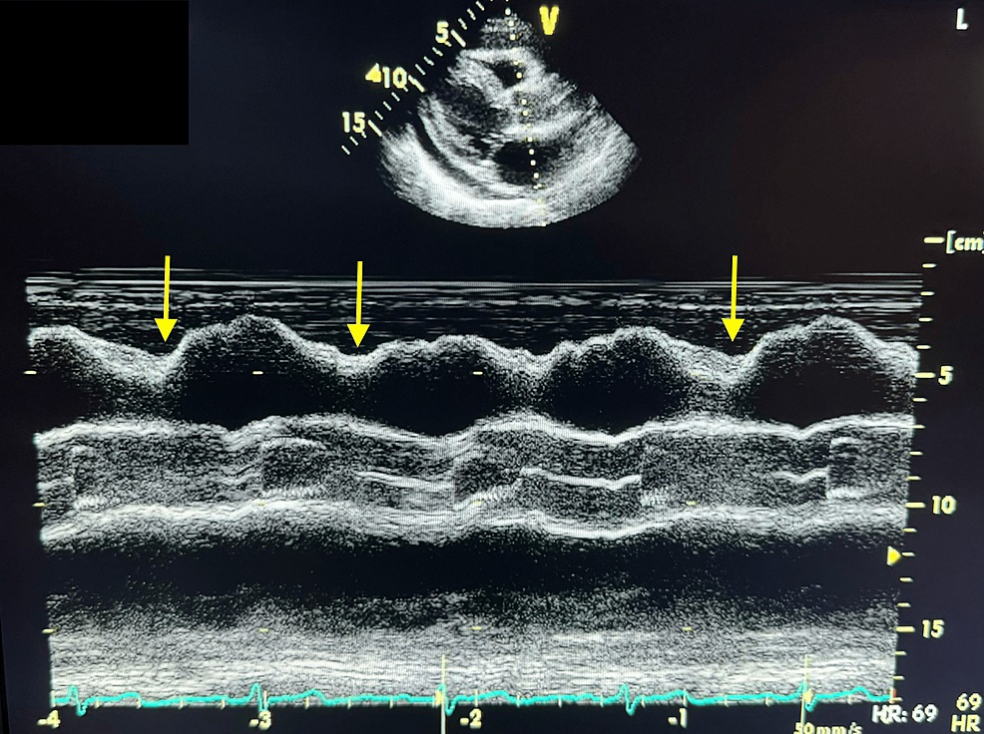
M-mode TTE from parasternal long-axis view showing evidence of right ventricular collapse during early diastole The yellow arrows denote the regions of right ventricular collapse during diastole. M-mode: motion mode; TTE: transthoracic echocardiogram

A 73-year-old male presented to an outpatient cardiologist for evaluation after experiencing five days of exertional dyspnea. The patient’s past medical history was significant for hypothyroidism, iron deficiency anemia, and chronic urinary retention secondary to lyme disease exposure. Transthoracic echocardiogram (TTE) revealed a moderate pericardial effusion with evidence of right atrial (Video 1, Figure 1) and right ventricular (Video 2, Figures 2-3) collapse. After discovery of effusion, the patient was transported by ambulance to a nearby tertiary care center for treatment.

**Video 1:** Apical view TTE video revealing pericardial effusion causing right atrial collapse TTE: Transthoracic echocardiogram

**Video 2:** Parasternal long-axis TTE video revealing right ventricular collapse as a result of pericardial effusion TTE: Transthoracic echocardiogram

Upon arrival at ED, the patient described his shortness of breath at its lowest severity. He was hemodynamically stable with blood pressure of 142/82 mm Hg and pulse of 66 BPM and showed no signs of respiratory distress with O2 saturation of 99% on room air. There were no signs of jugular venous distension (JVD), heart sounds were normal without murmurs, and the lungs were clear to auscultation bilaterally. TTE was repeated and confirmed the presence of a large circumferential pericardial effusion. Given the patient’s symptoms of shortness of breath and echocardiographic evidence of pericardial effusion with chamber collapse, the patient received an urgent pericardial window procedure. The pericardium was opened via a subxiphoid incision, and approximately 700 cc of extremely clear serous fluid was removed. Notably, the pericardium did not appear abnormal or inflamed. 

Postoperative laboratory testing (Table 1) revealed a significantly elevated thyroid-stimulating hormone (TSH) level of 39.66 µIU/ml and a low free thyroxine (FT4) level of 0.48 ng/dl. Labs were repeated one day later and revealed a TSH level of 50.31 µIU/ml, an FT4 level of 0.50 ng/dl, and a free triiodothyronine (FT3) level of 1.99 pg/ml. When questioning the patient regarding the use of thyroid medications, the patient revealed that he had refused using levothyroxine due to its synthetic composition. Instead he had been taking 1 grain of a desiccated thyroid extract once a day for approximately two weeks. He stated in the past that he had taken 3 grains of a different brand of desiccated thyroid extract that he was forced to stop when the supplement was discontinued by the manufacturer. It was unclear how long the patient had gone without taking thyroid supplements and why the medication was restarted at a dose of 1 grain once a day. The patient was seen by an inpatient endocrinologist and was counseled about the importance of using levothyroxine for the treatment of his hypothyroidism. Despite patient counseling and education, the patient refused the prescription of levothyroxine due to its synthetic composition and as a result was instructed to take 130 mg of a double compounded thyroid medication to be taken once a day and was discharged to home.

**Table 1:** Thyroid laboratory testing measured postoperatively revealing elevated TSH levels and low FT4 and FT3 levels TSH: Thyroid-stimulating hormone; FT4: free thyroxine; FT3: free triiodothyronine; postop: postoperative

Tests	Postop day 1	Postop day 2	Reference range
TSH (uIU/ml)	39.66	50.31	0.45-4.500
FT4 (ng/dl)	0.48	0.5	0.82-1.77
FT3 (pg/ml)	Not measured	1.99	2.0-4.4

## Discussion

Hypothyroidism is a common endocrine disorder that is characterized by low levels of thyroid hormones that affect multiple organ systems with widely varied clinical manifestations [6]. Thyroid hormones have a significant effect on myocardial contractility, total peripheral resistance, and heart rate with hypothyroidism commonly manifesting as sinus bradycardia, diastolic hypertension, and heart failure [7]. Pericardial effusion is another known complication of hypothyroidism that occurs as an increased permeability of epicardial vessels and decreased lymphatic drainage of albumin [5]. Progression to tamponade is rare as most pericardial effusions that occur as a result of hypothyroidism are small in size.

Cardiac tamponade most commonly presents with symptoms of chest pain, palpitations, and shortness of breath in addition to Beck’s triad of hypotension, JVD, and muffled heart sounds [8]. Interestingly, patients with cardiac tamponade as a result of hypothyroidism often present asymptomatically with atypical findings such as bradycardia or normal heart rate and elevated blood pressures [9]. Given this atypical presentation, as in our patient who presented only with mild dyspnea, the use of echocardiography is key in diagnosing cardiac tamponade and guiding treatment. Despite presenting hemodynamically stable, our patient showed evidence of right ventricular collapse during diastole which has been shown to be an important diagnostic clue in the progression of cardiac tamponade and impending hemodynamic instability [10,11]. 

Cardiac tamponade is a severe condition that often requires surgical care to alleviate symptoms and preserve heart function and reestablish hemodynamic stability. Surgical intervention is performed through the use of pericardiocentesis or a pericardial window technique in order to access the pericardium and drain fluid thus relieving strain on the heart [12,13]. Recognition of hypothyroidism as a cause of pericardial effusion and progression to tamponade is critical in guiding treatment and postoperative management. Patients with known hypothyroidism and pericardial effusion should be carefully monitored for progression to tamponade. Achieving a euthyroid state through optimal dosing of medication and strict medication adherence can significantly reduce the emergence of the condition as well as reoccurrence.

## Conclusions

This case report aims to highlight awareness of hypothyroidism as a cause of cardiac tamponade. Patients may present atypically compared to those experiencing tamponade from other underlying causes. Patients with a known history of hypothyroidism and pericardial effusions should be carefully monitored with periodic echocardiograms to recognize early signs of cardiac tamponade. Optimization of thyroid medications to achieve a euthyroid state is key in reducing the incidence of pericardial effusion and progression to cardiac tamponade.
